# Circulating MicroRNAs as Biomarkers of Prostate Cancer: The State of Play

**DOI:** 10.1155/2013/539680

**Published:** 2013-03-12

**Authors:** Nikhil Sapre, Luke A. Selth

**Affiliations:** ^1^Department of Surgery, Royal Melbourne Hospital, The University of Melbourne, Level 5 Clinical Sciences Building, Parkville, Melbourne, VIC 3050, Australia; ^2^Dame Roma Mitchell Cancer Research Laboratories, The University of Adelaide, P.O. Box 14, Rundle Mall, Adelaide, SA 5000, Australia

## Abstract

MicroRNAs are key regulators of gene expression and play critical roles in both normal physiology and pathology. Recent research has demonstrated that these molecules are present in body fluids, such as serum, plasma, and urine, and can be readily measured using a variety of techniques. More importantly, emerging evidence suggests that circulating or urine miRNAs are useful indicators of disease. Here, we consider the potential utility of such miRNAs as noninvasive biomarkers of prostate cancer, a disease that would benefit substantially from novel diagnostic and prognostic tools. The studies aimed at identifying diagnostic, prognostic, and/or predictive miRNAs for prostate cancer are summarised and reviewed. Finally, practical considerations that will influence the translation of this recent research into clinical implementation are discussed.

## 1. The Clinical Problem of Prostate Cancer

Prostate cancer is the second most common solid tumour in men worldwide and, despite significant advances in early diagnosis and management, it remains a leading cause of cancer-related death in men [1]. Pathological diagnosis of prostate cancer is usually obtained by a transrectal ultrasound-guided biopsy prompted by elevated levels of serum prostate-specific antigen (PSA) and/or an abnormal digital rectal examination (DRE). The use of PSA for the diagnosis of prostate cancer is associated with several clinical issues. PSA is not specific for this malignancy, being elevated in many other conditions including benign prostatic hyperplasia (BPH), urinary retention, prostatitis, trauma, and physical manipulation [2]. Moreover, elevated levels only correlate loosely with disease severity: approximately 30% of people with PSA 5–10 and >50% with PSA > 10 will have prostate cancer [3]. Conversely about 10–15% of people with PSA < 5 will harbour prostate cancer [4]. Perhaps more important than its diagnostic inaccuracy, three large clinical trials have revealed that PSA testing/screening is associated with a high rate of overdiagnosis and overtreatment [5–7].

Prostate cancer is characterised by distinctly unpredictable outcomes from latent, slow growing tumours to aggressive, rapidly lethal tumours. Although much effort has been put into finding biomarkers that would improve diagnosis, the pertinent clinical issue is the detection of aggressive forms of the disease at an early, curable stage. A significant proportion of cases follow an indolent course and may not require curative treatment. In fact, up to 40% of elderly men will harbour cancer within their prostates at autopsy [8, 9]. However, some cancers have the potential to metastasize and require aggressive, early clinical intervention. Unfortunately, current clinicopathological models do not allow clinicians to accurately discern between lethal and indolent prostate cancer at an early stage, leading to anxiety for both clinicians and patients about choosing the best treatment course [10]. Moreover, for the men who undergo an active surveillance regime for low-risk prostate cancer, it remains difficult to determine which patients will progress onto higher grade disease. This problem is compounded by the observation that disease grade may be misdiagnosed in up to 47% of cases [11]. Delaying curative treatment intervention in such patients could have lethal consequences.

Considering these issues, biomarkers that could improve diagnostic accuracy and better discriminate indolent from aggressive prostate cancers at an early stage would revolutionise the clinical management of this important disease. Moreover, identifying predictive biomarkers for the multitude of new treatment strategies being developed for metastatic prostate cancer [12] is of critical importance. In this paper, we will present evidence for the utility of circulating and urine miRNAs for such purposes.

## 2. MicroRNA Biogenesis and Function

MicroRNAs (miRNAs) are ~22 nucleotide-long, single-stranded, noncoding RNAs that were first reported in the nematode *Caenorhabditis elegans* [14]. Since that seminal finding, our understanding of miRNAs has increased substantially, and they are the best understood of the small RNAs today. The biogenesis of miRNAs has been comprehensively reported in many reviews (see, e.g., [15, 16]): briefly, long immature precursor miRNAs (pri-miRNAs) are transcribed by RNA polymerase II and processed in the nucleus by the RNase Drosha and nuclear protein Pasha (DGCR8) into 70–100 bp long pre-miRNAs [17]. Pre-miRNAs are exported to the cytoplasm by an Exportin 5-mediated mechanism where another RNase, Dicer, generates ~22 bp RNA duplexes [18, 19]. These dsRNAs comprise a mature miRNA guide strand (miR-5p) and a complementary passenger strand (miR-3p or miR*). The guide strand is preferentially incorporated into the RNA-induced silencing complex (RISC) and binds via partial complementarity to target sequences generally found within the 3′ UTRs of target mRNAs [20, 21]. The target mRNAs are subsequently degraded or, more commonly, repressed at a translational level [15].

It is currently estimated that the human genome encodes over 1800 distinct miRNAs (miRBase 19; [22]), which are estimated to regulate ~60% of all protein-coding genes [20]. A single miRNA can bind to multiple mRNAs and vice versa [23], creating a complex and widespread network of miRNA:mRNA interactions that can profoundly influence gene expression programs. The importance of miRNAs is evidenced by their critical functions in essentially all normal physiological processes, including cell cycle processes, development, survival, differentiation, growth, apoptosis, and the immune response [15].

## 3. MicroRNA Dysregulation in Cancer

Given their physiological importance, it is not surprising that miRNAs also play important roles in the genesis and progression of cancer. This concept was first demonstrated by Calin and colleagues, who found that a genomic region at 13q14 containing two miRNAs (miR-15a and miR-16-1) is frequently deleted in leukemia [24]. Since then, the dysregulation of miRNA expression has been demonstrated in all types of human neoplasm. Aberrantly expressed miRNAs function in cancer by targeting relevant coding mRNAs: oncomiRNAs target genes that inhibit malignancy while tumour suppressor miRNAs target oncogenes [25]. It is important to recognise that these regulatory factors can have dichotomous functions in different tumours—acting to promote malignancy in some and to inhibit malignancy in others—based on tumour-specific expression patterns of miRNAs and their target genes.

Mechanisms by which miRNA function is altered in cancer include deletion/amplification of miRNA genes, modulation of miRNA gene expression through epigenetic mechanisms or dysregulation of transcription factors, and mutation of miRNA loci or their target sequences [26]. Dysregulated miRNAs can impact on many different aspects of the genesis and progression of cancer, including proliferation, metastasis (local invasion and colonisation), apoptosis, and angiogenesis, amongst others (for review, see [27]). In addition, aberrations in miRNA processing can also modulate miRNA function in cancer: indeed, such defects are a common feature of malignancy. For example, Dicer was shown to be downregulated in lung cancer and associated with reduced postoperative survival [28]. Moreover, silencing of Dicer in murine lung tissue enhanced the development of lung cancer [29]. Dysregulation of genes coding for Argonaute proteins, which are critical elements of the RISC complex, has also been observed in a variety of malignancies including Wilms tumours [30], colon cancer [31], and testicular cancer [32].

## 4. MicroRNAs as Biomarkers of Disease

The realisation that miRNAs are deregulated in human cancers has generated considerable interest with regard to their potential as biomarkers. miRNAs have a number of desirable characteristics for such an application. Perhaps most importantly, miRNA expression profiles are often tissue, developmental, and disease specific. For example, early work demonstrated that miRNA expression signatures accurately distinguished between different tumour types and could accurately identify cancers of histologically uncertain origin [33]. Importantly, in this study the miRNA signatures were considerably more useful than equivalent mRNA signatures. miRNA profiles have also been used to subtype several cancer types, including breast and ovarian [34, 35]. Since those seminal studies, the utility of miRNA expression profiles to identify and stratify cancer has become increasingly evident (for review, see [25]). Other useful attributes of miRNAs for biomarker applications include their exceptional stability in various types of clinical samples, including formalin-fixed paraffin embedded tissues [36], ease of quantitation using PCR-based assays, and conservation between species [37], which may facilitate the use of animal models of cancer for biomarker discovery.

Recent research has shown that miRNAs possess one additional feature of an ideal biomarker, namely, an ability to be sampled noninvasively. In 2008, a number of groups reported the presence of circulating miRNAs in cell-free fractions of blood (i.e., serum and plasma) and presented evidence suggesting that a subset of these molecules could be useful indicators of disease [36, 38, 39]. Many studies have since shown that circulating miRNAs are associated with various malignancies and may be applied as diagnostic, prognostic, and predictive tools (for review, see [27, 40]). Circulating miRNAs are remarkably stable, resisting degradation by ribonucleases and severe physicochemical conditions such as boiling, extended storage, freeze-thawing, and extreme pH levels [36, 41], a characteristic that will facilitate their translation to clinical applications. This stability is due to the presence of protective structures including proteins (e.g., Ago2), exosomes, microvesicles, and lipoprotein, which bind or encapsulate cell-free miRNAs in the blood [42–45]. It is likely that protection of circulating miRNAs facilitates their ability to regulate gene expression in recipient cells [46, 47]—we have previously termed such mobile miRNAs “hormomirs” because of their potential hormone-like characteristics [40]. MiRNAs have now been isolated from a range of body fluids, greatly expanding their clinical potential [48–50]. In a study of 12 different body fluids, the number of detectable miRNAs ranged from 204 (urine) to 458 (saliva), indicating that they represent a rich and diverse source of potential biomarkers [48].

The potential of miRNAs derived from body fluids as noninvasive biomarkers for different tumor entities has been discussed extensively in recent review articles (see, e.g., [46, 63]). Here, we will specifically discuss the application of circulating (serum and plasma) and urine miRNAs for detection and management of prostate cancer.  Table 1 summarises the studies that are relevant to this topic. 

### 4.1. Circulating (Serum and Plasma) miRNAs as Biomarkers of Prostate Cancer

The Tewari laboratory was the first to demonstrate an association between a circulating miRNA and prostate cancer [36]. In this pioneering study, a mouse xenograft system was used to identify tumour-derived miRNAs in plasma. One of these, miR-141, was found to accurately differentiate between men with castration-resistant prostate cancer (CRPC) and healthy men (area under the receiver operating characteristic curve (AUC) = 0.907).

A number of studies have assessed the potential of circulating miRNAs to diagnose early-stage, localised prostate cancer. Moltzahn and colleagues compared circulating miRNA profiles in men with early-stage prostate cancer and healthy men [52] using a novel microfluidics-based multiplex qRT-PCR platform. Of the 10 miRNAs significantly altered in the malignant samples, miR-106a and miR-1274 possessed the best diagnostic capability (AUC for each = 0.928). An approach targeting known prostate cancer-associated miRNAs found that miR-21 and miR-221 were elevated in the plasma of men with localized cancer compared to healthy controls [55]. Bryant and colleagues assessed the diagnostic capacity of plasma miRNAs using Exiqon's high-throughput qRT-PCR platform [57]. Twelve miRNAs were differentially quantitated in the circulation of men with prostate cancer (of diverse grade and stage) compared to healthy men, with miR-107 showing the greatest fold change. MiR-107 had an AUC of 0.62 compared to an AUC of 0.79 for PSA. Most recently, profiling using an Illumina microarray platform led to the identification of 5 miRNAs—let-7c, let-7e, miR-30c, miR-622, and miR-1285—with diagnostic capacity [59]. A signature of the plasma levels of these miRNAs could differentiate prostate cancer from BPH and healthy controls with an AUC of 0.924 and 0.860, respectively.

Other studies have focussed on identifying miRNAs associated with metastatic disease that could be applied as prognostic markers at the time of diagnosis or to detect recurrence following primary treatment. Our group utilized the TRansgenic Adenocarcinoma of Mouse Prostate (TRAMP) model to discover circulating miRNAs that demarcated mice with advanced disease from healthy mice [37]. Four of the TRAMP-associated miRNAs—miR-141, miR-298, miR-346, and miR-375—were subsequently shown to be elevated in sera from patients with metastatic castration resistant prostate cancer, and the intratumoural expression of miR-375 was inversely associated with biochemical recurrence in men treated by radical prostatectomy. This study was the first to demonstrate that certain serum miRNAs are common between human and murine forms of prostate cancer, highlighting the utility of mouse models for this research. Three studies compared circulating miRNAs from men with localised or metastatic cancer by Taqman multiplexed qRT-PCR [53, 57, 58]. In validation cohorts, both miR-141 and miR-375 were found to be markers of systemic disease in each of the three studies. Moreover, these miRNAs were associated with tumour stage and Gleason score in serum samples taken from men with localized disease immediately prior to RP [53]. Two other studies compared serum/plasma collected prior to RP from men with different clinicopathological parameters (i.e., Gleason score, tumour stage, cancer of the prostate risk assessment (CAPRA) score, and D'Amico score) in an effort to identify prognostic miRNAs [52, 61]. A number of miRNAs were associated with such clinicopathological parameters (Table 1), but none were consistent between the two studies.

The utility of circulating miRNAs as predictors of treatment response is a particularly exciting concept. Although the application of circulating miRNAs for this purpose is in its infancy, two recent studies have provided evidence for their potential in prostate cancer. Zhang and colleagues measured miR-21 levels in patients with localised and metastatic prostate cancer and found that this miRNA was significantly higher in CRPC patients who exhibited resistance to the chemotherapeutic docetaxel [64]. While the sample size in this study was small, it nevertheless represents an important finding that warrants further investigation. A more recent study specifically aimed to assess the utility of plasma miR-141 as a biomarker of treatment response in patients with metastatic prostate cancer receiving chemotherapy, hormone therapy, or novel agents such as vaccines and kinase inhibitors [54]. When assessing the cohort as a whole, miR-141 had a sensitivity of 78.9% and specificity of 68.8% in predicting clinical progression.

### 4.2. Urine miRNAs as Biomarkers of Prostate Cancer

For physiological and anatomical reasons, urine may represent a valuable source of miRNA biomarkers for urological cancers. Bryant and colleagues were the first to test this concept [57]: in this study, 5 known prostate cancer-associated miRNAs (miR-107, miR-141, miR-200b, miR-375, and miR-574-3p) were measured in the urine of prostate cancer patients (*n* = 118) and healthy controls (*n* = 17). The patients underwent a digital rectal examination (DRE) before a first pass sample enriched with prostate cells was obtained. All of the candidate miRNAs were detectable in urine, but only miR-107 (*P* = 0.001, AUC = 0.74) and miR-574-3p (*P* = 0.012, AUC = 0.66) were at differential levels in men with prostate cancer. In this cohort, the diagnostic value of these miRNAs was greater than PCA3 mRNA, a urine marker of prostate cancer [65] that has been incorporated into an FDA-approved test (http://www.gen-probe.com/products-services/progensa-pca3).

Collectively, the studies described above suggest that circulating miRNAs may assist in the diagnosis, prognosis, and prediction of prostate cancer. Unfortunately, there is little agreement between most of these studies: some of the reasons that may underlie these conflicting results are discussed below. One positive finding is the robust association of miR-141 and miR-375 with metastatic disease [36, 37, 53–55, 57, 58]. These miRNAs could potentially be applied in a number of clinical situations: at the time of diagnosis, to identify patients with undetected micrometastases or tumours likely to metastasise who would benefit from more aggressive therapeutic strategies; following primary treatment, to identify metastatic relapse; and to monitor treatment response in advanced disease. In addition, it seems very likely that these miRNAs are tumour derived [37, 66]: gaining a better understanding of their function in normal tissues and potential involvement in prostate cancer progression is vital. While we believe that circulating miR-141 and miR-375 have significant potential as novel prognostic biomarkers of prostate cancer, it must be appreciated that both of these molecules have been associated with other pathologies [67, 68]. Such a lack of disease specificity likely applies to other key miRNAs in circulation. Utilising miRNA signatures rather than measuring single miRNAs should adequately address this issue.

### 4.3. Important Considerations for Quantitating Circulating and Urine miRNAs

**Figure 1:**
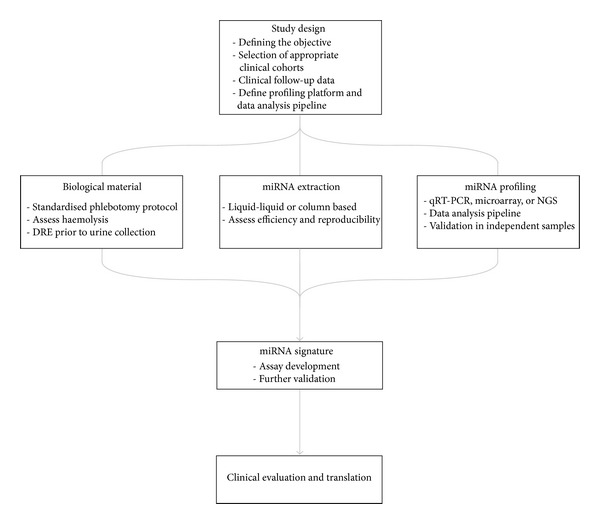
Pipeline for developing circulating and urine miRNAs as biomarkers of disease, with important considerations shown. Study design is critical and will influence all aspects of the methodology. Clinical evaluation and translation, including clinical trials, commercialisation, and approval, have been discussed elsewhere (e.g., see [13]) and are not included in this paper.

Many factors are likely to impact on our ability to identify *bona fide* and clinically relevant miRNA markers of disease in circulation and urine. In Figure 1, we have integrated these factors into a concise miRNA biomarker discovery pipeline. 

#### 4.3.1. Sampling the Biological Material

Robust, standardised methodology for sampling the biological material is critically important. For example, contamination of blood fluids with intact or lysed (i.e., haemolysis) blood cells during phlebotomy and sample processing can have a profound effect on the resultant miRNA profile [69–71]. Performing an additional centrifugation step after plasma/serum preparation is likely to remove the majority of intact cellular material [69, 70]. To estimate the extent of haemolysis, one can measure free haemoglobin or certain miRNAs that are highly expressed in red and white blood cells (e.g., miR-15b, miR-16, and miR-451) [70, 71]. This may allow the removal of outlier samples with high levels of cellular content.

Similarly, although miRNA profiling from urine is in its infancy, it is reasonable to suggest that the sampling strategy will have a significant impact on the measurable miRNA milieu. Urine samples should ideally be taken as first pass samples immediately after a DRE to enrich urine sample for prostate cells. The commercially available urine-based PCA3 test for prostate cancer is normally performed after a modified DRE (3 strokes per lobe). A study aimed at analysing performance characteristics of the PCA3 test found that, in the absence of a DRE, an unacceptably low number of patients (75.9% versus 96.7% following DRE) had sufficient prostate cells in their urine for robust measurement [72]. Of note, this study found that the PCA3 score was independent of the type of DRE procedure (normal DRE versus 3 strokes per lobe versus 8 strokes per lobe).

#### 4.3.2. MicroRNA Extraction

Many different protocols for isolating miRNAs from serum/plasma and urine have been developed. In general, these protocols comprise guanidinium-phenol (Trizol, Qiazol, etc.) extraction of the sample followed by purification of miRNAs using either alcohol-mediated precipitation or column-based methods [48, 73]. A recent study suggested that a standard liquid-liquid Trizol extraction method may result in better recovery and decreased intra-assay variance than Invitrogen mirVana columns [70]. We have also noted increased recovery of miRNAs using liquid-liquid phenol extraction compared to Qiagen's miRNeasy columns (L. A. Selth, unpublished observations), although the increased hands-on time required for the former offset its possible benefits. In the past two years, a number of commercial kits designed specifically for the extraction of miRNAs from serum, plasma, urine, and exosomes have entered the market as the volume of research in this area has increased. Unfortunately, a robust comparison of commercial- and laboratory-developed *ad hoc *purification strategies is lacking.

#### 4.3.3. MicroRNA Profiling

Measuring specific miRNAs or profiling the complete miRNA population is generally achieved using either qRT-PCR, microarrays, or next-generation sequencing (NGS). Of these, by far the most commonly employed is qRT-PCR, probably because of its increased sensitivity and accuracy. Microarrays and NGS are less sensitive but can profile many more miRNAs. Moreover, NGS has the ability to identify previously unknown miRNAs that would not be amplified by qRT-PCR or anneal to microarray chips. Given that the miRNAome is likely to expand further and the emerging notion that miRNA 5′- and 3′-end structural variants, termed isomirs, are commonly expressed and have been linked to cancer [74, 75], this represents a significant advantage.

Each of the three aforementioned methodologies is associated with a number of unresolved issues. Arguably, the most important of these is how to best normalise miRNA measurements to account for biological and technical variability. Quantitation of small RNAs extracted from 50–400 *μ*L serum/plasma using spectrophotometry is, in our hands, not possible. Moreover, suitable reference genes in serum/plasma/urine have not been identified: studies of colorectal cancer and lymphoma utilised miR-16 for normalisation purposes [39, 76], but the utility of this miRNA for prostate cancer has been called into question [56]. Moreover, miR-16 is highly expressed in erythrocytes and can therefore be heavily influenced by haemolysis [70, 71]. Sanders and colleagues recently evaluated a series of reference small RNAs in prostate cancer, bladder cancer, and renal cell carcinoma and found *SNORD43 *to be a stable reference gene for all three malignancies [62]. However, the suitability of *SNORD43* has yet to be validated by other groups or in other prostate cancer cohorts. To overcome these issues, a number of guidelines have been devised. First, most protocols recommend a constant starting volume of serum/plasma/urine. Second, correcting for technical variability can be achieved by spiking in synthetic nonhuman miRNAs (i.e., cel-miR-39) [36, 73]. This latter guideline most commonly applies to experiments in which selected candidate miRNAs are being quantitated by qRT-PCR. Profiling experiments in which the entire or large subsets of the miRNAome are measured by qRT-PCR, microarray, or NGS afford other opportunities for normalisation. Since data is obtained for hundreds to thousands of miRNAs, normalisation methods that utilise all or most of the data points, including median, quantile, Loess, and global, can be applied [77]. These methods are likely to correct more robustly than endogenous or spiked-in controls. Comparisons of different normalisation methods for high-throughput qRT-PCR (e.g., Taqman low-density PCR arrays) and microarrays have been performed (e.g., [78–80]). The method of choice can have a significant impact on the discovery of “differentially expressed” miRNAs and should be carefully considered.

Two other issues in the profiling/data processing phase must be taken into account. First, it is important to use false discovery rate (FDR) correction when profiling large numbers of miRNAs with any of the methods described above. Second, it is strongly advised that differentially expressed miRNAs identified by microarrays or NGS are validated by qRT-PCR, a more sensitive and accurate technique. Both of these factors are likely to reduce false positive and other erroneous discoveries and result in more robust disease-associated signatures.

Finally, it is worth highlighting that urine miRNA concentrations can differ significantly based on the hydration status of patients. Whilst the gold standard to account for such differences is to measure 24-hour urine volumes, a more feasible method may be to normalise expression data to urinary osmolarity or specific gravity.

#### 4.3.4. Study Design and Further Validation

Even if the experimental workflow (comprising collection of the biomaterial, extraction, miRNA profiling, and data processing) is robust, the experiment can be impaired by a poor study design. This factor is probably a major reason why very few markers can be validated in further studies. A number of factors need to be considered with the complete biomarker development pipeline firmly in mind [13, 81]. First, cohort selection is critical. Future studies aimed at identifying diagnostic miRNAs in circulation/urine should focus on clinically relevant groups (e.g., PSA- or non-PSA screened, biopsied or not), while studies assessing the prognostic potential of miRNAs require cohorts with long-term clinical followup. Second, the design and analysis strategy for these types of studies should be determined in light of their overall objectives. Most studies use “class discovery” or “class prediction” to identify clinically relevant miRNA biomarkers, and these objectives must be understood and factored into the experimental design [82]. Finally, validation in independent sample sets is vitally important: only a small proportion of the studies conducted so far in prostate cancer patients have adhered to this guideline (Table 1).

## 5. Concluding Remarks

While there is genuine potential for circulating and urine miRNAs in diagnostic, prognostic, and predictive applications, clinical implementation of a noninvasive miRNA test for prostate cancer is still a distant goal. The studies that have been conducted thus far are heterogeneous in terms of objectives and methodology, which have often yielded conflicting data and outcomes. Improving the consistency and standardisation of these factors is of critical importance. Moreover, cohorts with long clinical followup to validate some of the promising findings, such as the association between miR-141 and miR-375 and metastasis, are yet to be analysed. Despite these challenges, and in light of the fact that circulating miRNAs were discovered just 4 years ago, we believe that the outlook in this field is bright.

## Figures and Tables

**Table 1:** Studies investigating the potential of circulating miRNAs as biomarkers of prostate cancer.

Body fluid	Sample size	Methodology	Key findings	Reference
Plasma	25 patients (metastatic PCa), 25 healthy controls	qRT-PCR (6 miRNAs)	miR-141 levels could differentiate metastatic PCa patients from healthy subjects	Mitchell et al., 2008 [36]

Serum	6 patients (stages 2–4 PCa), 8 healthy controls	Microarray (custom) (547 miRNAs)	15 miRNAs were elevated in PCa patients. However, serum miRNAs could not distinguish between different cancer types	Lodes et al., 2009 [51]

Serum	56 patients (20 localized PCa, 20 androgen-dependent PCa, 10 CRPC^2^), 6 BPH^3^ controls	qRT-PCR (miR-21 only)	miR-21 was elevated in CRPC patients compared to BPH and associated with resistance to docetaxel in CRPC patients	Zhang et al., 2010 [47]

Serum	29 patients (9 low risk, 11 intermediate risk, and 9 high risk)^1^, 9 healthy controls	qRT-PCR (384 miRNAs)	10 miRNAs were altered in PCa patients compared to healthy controls. 7 miRNAs were correlated with different risk groups.	Moltzahn et al., 2011 [52]

Serum	Profiling: 7 high grade, 14 low grade patients. Validation: 116 patients (various grades)	qRT-PCR (667 miRNAs)	miR-141, miR-200b, and miR-375 were elevated in serum from high-grade patients and correlated with clinicopathological parameters	Brase et al., 2011 [53]

Plasma	21 patients (metastatic PCa)	qRT-PCR (miR-141 only)	miR-141 levels were associated with clinical progression and positively correlated with prostate specific antigen	Gonzales et al., 2011 [54]

Plasma	51 patients (18 localized PCa, 8 local advanced, and 25 metastatic), 20 healthy controls	qRT-PCR (miR-21, miR-141, and miR-221)	miR-21 and miR-221 levels were elevated in PCa patients compared to healthy controls. miR-21, miR-141, and miR-221 levels were higher in metastatic compared to localised tumours	Agaoglu et al., 2011 [55]

Serum	45 patients (37 localized PCa, 8 metastatic), 18 BPH controls, and 20 healthy controls	qRT-PCR (5 miRNAs)	miR-26a, miR-195, and let-7i levels were elevated in PCa compared to BPH samples	Mahn et al., 2011 [56]

Serum	Profiling: 14 TRAMP mice, 14 healthy controls. Validation: 25 patients (metastatic CRPC), 25 healthy controls	Microarray (Affymetrix; 609 murine miRNAs), qRT-PCR (10 human miRNAs)	miR-141, miR-298, miR-346, and miR-375 levels were elevated in metastatic CRPC compared to healthy controls. Expression of miR-375 in primary tumours was associated with biochemical relapse	Selth et al., 2012[37]

Plasma, serum, and urine	Profiling: 78 patients (various grades, 15 with diagnosed metastases), 28 healthy controls. Validation: 119 patients (47 recurrent after RP^4^, 72 nonrecurrent)	qRT-PCR (742 miRNAs)	12 circulating miRNAs were at altered levels in PCa patients compared to healthy controls. 16 circulating miRNAs were at altered levels in metastatic versus localised PCa (including miR-141 and miR-375). Urinary levels of miR-107 and miR-574-3p exhibited significant diagnostic value	Bryant et al., 2012 [57]

Serum	84 patients (28 low risk localised disease, 30 high risk localised disease, and 26 metastatic CRPC	qRT-PCR (667 miRNAs)	miR-375, miR-141, miR-378, and miR-409-3p were at altered levels in metastatic CRPC compared localised cancer	Nguyen et al., 2013 [58]

Plasma	Profiling: 25 patients (localised and metastatic PCa), 17 BHP controls. Validation: 80 patients (localised and metastatic PCa), 44 BHP controls, and 54 healthy controls	Microarray (Illumina; 1146 miRNAs), qRT-PCR (8 miRNAs) (609 murine miRNAs, 10 human miRNAs)	5 miRNAs with significant diagnostic value were identified (let-7c, let-7e, miR-30c, miR-622, and miR-1285)	Chen et al., 2012 [59]

Plasma	23 patients (15 androgen dependent PCa, 8 CRPC), 20 healthy controls	qRT-PCR (miR-221 only)	miR-221 was elevated in PCa patients compared to healthy controls, and higher in androgen-dependent PCa compared to CRPC	Zheng et al., 2012 [60]

Plasma	82 patients (various risk scores^1,5^)	qRT-PCR (5 miRNAs)	miR-20a, miR-21, miR-145, and miR-221 were associated with tumour risk scores^1,5^	Shen et al., 2012 [61]

Serum	72 patients (24 localised prostate cancer, 24 bladder cancer, and 24 renal cell carcinoma), 48 noncancer controls	qRT-PCR (8 putative reference small RNAs and miR-21)	*SNORD43 *may be a suitable reference gene for the analysis of circulating miRNA in patients with urological malignancies	Sanders et al., 2012 [62]

^1^“Cancer of the prostate risk assessment” score

^2^Castration-resistant prostate cancer

^3^Benign prostatic hyperplasia

^4^Radical prostatectomy

^5^D'Amico score

## References

[1] Bray F, Ren J-S, Masuyer E, Ferlay J (2013). Global estimates of cancer prevalence for 27 sites in the adult population in 2008. *International Journal of Cancer*.

[2] Oesterling JE (1991). Prostate specific antigen: a critical assessment of the most useful tumor marker for adenocarcinoma of the prostate. *Journal of Urology*.

[3] Catalona WJ, Smith DS, Ratliff TL (1991). Measurement of prostate-specific antigen in serum as a screening test for prostate cancer. *The New England Journal of Medicine*.

[4] Thompson IM, Pauler DK, Goodman PJ (2004). Prevalence of prostate cancer among men with a prostate-specific antigen level ≤ 4.0 ng per milliliter. *The New England Journal of Medicine*.

[5] Hugosson J, Carlsson S, Aus G (2010). Mortality results from the Göteborg randomised population-based prostate-cancer screening trial. *The Lancet Oncology*.

[6] Schröder FH, Hugosson J, Roobol MJ (2009). Screening and prostate-cancer mortality in a randomized european study. *The New England Journal of Medicine*.

[7] Andriole GL, Crawford ED, Grubb RL (2009). Mortality results from a randomized prostate-cancer screening trial. *The New England Journal of Medicine*.

[8] Sánchez-Chapado M, Olmedilla G, Cabeza M, Donat E, Ruiz A (2003). Prevalence of prostate cancer and prostatic intraepithelial neoplasia in Caucasian Mediterranean males: an autopsy study. *Prostate*.

[9] Stamatiou K, Alevizos A, Agapitos E, Sofras F (2006). Incidence of impalpable carcinoma of the prostate and of non-malignant and precarcinomatous lesions in Greek male population: an autopsy study. *Prostate*.

[10] Albertsen PC, Hanley JA, Gleason DF, Barry MJ (1998). Competing risk analysis of men aged 55 to 74 years at diagnosis managed conservatively for clinically localized prostate cancer. *Journal of the American Medical Association*.

[11] Mufarrij P, Sankin A, Godoy G, Lepor H (2010). Pathologic outcomes of candidates for active surveillance undergoing radical prostatectomy. *Urology*.

[12] Attard G, De Bono JS (2011). Translating scientific advancement into clinical benefit for castration-resistant prostate cancer patients. *Clinical Cancer Research*.

[13] Phillips KA, Van Bebber S, Issa AM (2006). Diagnostics and biomarker development: priming the pipeline. *Nature Reviews Drug Discovery*.

[14] Lee RC, Feinbaum RL, Ambros V (1993). The C. elegans heterochronic gene lin-4 encodes small RNAs with antisense complementarity to lin-14. *Cell*.

[15] Bartel DP (2009). MicroRNAs: target recognition and regulatory functions. *Cell*.

[16] Winter J, Jung S, Keller S, Gregory RI, Diederichs S (2009). Many roads to maturity: microRNA biogenesis pathways and their regulation. *Nature Cell Biology*.

[17] Lee Y, Ahn C, Han J (2003). The nuclear RNase III Drosha initiates microRNA processing. *Nature*.

[18] Lund E, Güttinger S, Calado A, Dahlberg JE, Kutay U (2004). Nuclear export of microRNA precursors. *Science*.

[19] Yi R, Qin Y, Macara IG, Cullen BR (2003). Exportin-5 mediates the nuclear export of pre-microRNAs and short hairpin RNAs. *Genes and Development*.

[20] Friedman RC, Farh KKH, Burge CB, Bartel DP (2009). Most mammalian mRNAs are conserved targets of microRNAs. *Genome Research*.

[21] Lewis BP, Burge CB, Bartel DP (2005). Conserved seed pairing, often flanked by adenosines, indicates that thousands of human genes are microRNA targets. *Cell*.

[22] Kozomara A, Griffiths-Jones S (2011). MiRBase: integrating microRNA annotation and deep-sequencing data. *Nucleic Acids Research*.

[23] Zhang R, Su B (2009). Small but influential: the role of microRNAs on gene regulatory network and 3′UTR evolution. *Journal of Genetics and Genomics*.

[24] Calin GA, Dumitru CD, Shimizu M (2002). Frequent deletions and down-regulation of micro-RNA genes miR15 and miR16 at 13q14 in chronic lymphocytic leukemia. *Proceedings of the National Academy of Sciences of the United States of America*.

[25] Croce CM (2009). Causes and consequences of microRNA dysregulation in cancer. *Nature Reviews Genetics*.

[26] Schaefer A, Stephan C, Busch J, Yousef GM, Jung K (2010). Diagnostic, prognostic and therapeutic implications of microRNAs in urologic tumors. *Nature Reviews Urology*.

[27] Ma R, Jiang T, Kang X (2012). Circulating microRNAs in cancer: origin, function and application. *Journal of Experimental and Clinical Cancer Research*.

[28] Karube Y, Tanaka H, Osada H (2005). Reduced expression of Dicer associated with poor prognosis in lung cancer patients. *Cancer Science*.

[29] Kumar MS, Pester RE, Chen CY (2009). Dicer1 functions as a haploinsufficient tumor suppressor. *Genes and Development*.

[30] Koesters R, Adams V, Betts D (1999). Human eukaryotic initiation factor EIF2C1 gene: cDNA sequence, genomic organization, localization to chromosomal bands 1p34-p35, and expression. *Genomics*.

[31] Li L, Yu C, Gao H, Li Y (2010). Argonaute proteins: potential biomarkers for human colon cancer. *BMC Cancer*.

[32] Qiao D, Zeeman AM, Deng W, Looijenga LHJ, Lin H (2002). Molecular characterization of hiwi, a human member of the piwi gene family whose overexpression is correlated to seminomas. *Oncogene*.

[33] Lu J, Getz G, Miska EA (2005). MicroRNA expression profiles classify human cancers. *Nature*.

[34] Iorio MV, Ferracin M, Liu CG (2005). MicroRNA gene expression deregulation in human breast cancer. *Cancer Research*.

[35] Iorio MV, Visone R, Di Leva G (2007). MicroRNA signatures in human ovarian cancer. *Cancer Research*.

[36] Mitchell PS, Parkin RK, Kroh EM (2008). Circulating microRNAs as stable blood-based markers for cancer detection. *Proceedings of the National Academy of Sciences of the United States of America*.

[37] Selth LA, Townley S, Gillis JL (2012). Discovery of circulating microRNAs associated with human prostate cancer using a mouse model of disease. *International Journal of Cancer*.

[38] Chim SSC, Shing TKF, Hung ECW (2008). Detection and characterization of placental microRNAs in maternal plasma. *Clinical Chemistry*.

[39] Lawrie CH, Gal S, Dunlop HM (2008). Detection of elevated levels of tumour-associated microRNAs in serum of patients with diffuse large B-cell lymphoma. *British Journal of Haematology*.

[40] Selth LA, Tilley WD, Butler LM (2012). Circulating microRNAs: macro-utility as markers of prostate cancer?. *Endocrine-Related Cancer*.

[41] Chen X, Ba Y, Ma L (2008). Characterization of microRNAs in serum: a novel class of biomarkers for diagnosis of cancer and other diseases. *Cell Research*.

[42] Arroyo JD, Chevillet JR, Kroh EM (2011). Argonaute2 complexes carry a population of circulating microRNAs independent of vesicles in human plasma. *Proceedings of the National Academy of Sciences of the United States of America*.

[43] Valadi H, Ekström K, Bossios A, Sjöstrand M, Lee JJ, Lötvall JO (2007). Exosome-mediated transfer of mRNAs and microRNAs is a novel mechanism of genetic exchange between cells. *Nature Cell Biology*.

[44] Vickers KC, Palmisano BT, Shoucri BM, Shamburek RD, Remaley AT (2011). MicroRNAs are transported in plasma and delivered to recipient cells by high-density lipoproteins. *Nature Cell Biology*.

[45] Skog J, Würdinger T, van Rijn S (2008). Glioblastoma microvesicles transport RNA and proteins that promote tumour growth and provide diagnostic biomarkers. *Nature Cell Biology*.

[46] Cortez MA, Bueso-Ramos C, Ferdin J, Lopez-Berestein G, Sood AK, Calin GA (2011). MicroRNAs in body fluids-the mix of hormones and biomarkers. *Nature Reviews Clinical Oncology*.

[47] Zhang Y, Liu D, Chen X (2010). Secreted monocytic miR-150 enhances targeted endothelial cell migration. *Molecular Cell*.

[48] Weber JA, Baxter DH, Zhang S (2010). The microRNA spectrum in 12 body fluids. *Clinical Chemistry*.

[49] Hanke M, Hoefig K, Merz H (2010). A robust methodology to study urine microRNA as tumor marker: microRNA-126 and microRNA-182 are related to urinary bladder cancer. *Urologic Oncology*.

[50] Park NJ, Zhou H, Elashoff D (2009). Salivary microRNA: discovery, characterization, and clinical utility for oral cancer detection. *Clinical Cancer Research*.

[51] Lodes MJ, Caraballo M, Suciu D, Munro S, Kumar A, Anderson B (2009). Detection of cancer with serum miRNAs on an oligonucleotide microarray. *PLoS ONE*.

[52] Moltzahn F, Olshen AB, Baehner L (2011). Microfluidic-based multiplex qRT-PCR identifies diagnostic and prognostic microRNA signatures in the sera of prostate cancer patients. *Cancer Research*.

[53] Brase JC, Johannes M, Schlomm T (2011). Circulating miRNAs are correlated with tumor progression in prostate cancer. *International Journal of Cancer*.

[54] Gonzales JC, Fink LM, Goodman Jr. OB, Symanowski JT, Vogelzang NJ, Ward DC (2011). Comparison of circulating MicroRNA 141 to circulating tumor cells, lactate dehydrogenase, and prostate-specific antigen for determining treatment response in patients with metastatic prostate cancer. *Clinical Genitourinary Cancer*.

[55] Agaoglu FY, Kovancilar M, Dizdar Y (2011). Investigation of miR-21, miR-141, and miR-221 in blood circulation of patients with prostate cancer. *Tumour Biology*.

[56] Mahn R, Heukamp LC, Rogenhofer S, Von Ruecker A, Müller SC, Ellinger J (2011). Circulating microRNAs (miRNA) in serum of patients with prostate cancer. *Urology*.

[57] Bryant RJ, Pawlowski T, Catto JWF (2012). Changes in circulating microRNA levels associated with prostate cancer. *British Journal of Cancer*.

[58] Nguyen HCN, Xie W, Yang M (2013). Expression differences of circulating microRNAs in metastatic castration resistant prostate cancer and low-risk, localized prostate cancer. *Prostate*.

[59] Chen Z-H, Zhang G-L, Li H-R (2012). A panel of five circulating microRNAs as potential biomarkers for prostate cancer. *Prostate*.

[60] Zheng C, Yinghao S, Li J (2012). MiR-221 expression affects invasion potential of human prostate carcinoma cell lines by targeting DVL2. *Medical Oncology*.

[61] Shen J, Hruby GW, McKiernan JM (2012). Dysregulation of circulating microRNAs and prediction of aggressive prostate cancer. *Prostate*.

[62] Sanders I, Holdenrieder S, Walgenbach-Brünagel G (2012). Evaluation of reference genes for the analysis of serum miRNA in patients with prostate cancer, bladder cancer and renal cell carcinoma. *International Journal of Urology*.

[63] Brase JC, Wuttig D, Kuner R, Sültmann H (2010). Serum microRNAs as non-invasive biomarkers for cancer. *Molecular Cancer*.

[64] Zhang HL, Yang LF, Zhu Y (2011). Serum miRNA-21: elevated levels in patients with metastatic hormone-refractory prostate cancer and potential predictive factor for the efficacy of docetaxel-based chemotherapy. *Prostate*.

[65] Hessels D, Klein Gunnewiek JMT, Van Oort I (2003). DD3^PCA3^-based molecular urine analysis for the diagnosis of prostate cancer. *European Urology*.

[66] Szczyrba J, Löprich E, Wach S (2010). The microRNA profile of prostate carcinoma obtained by deep sequencing. *Molecular Cancer Research*.

[67] Cheng H, Zhang L, Cogdell DE (2011). Circulating plasma MiR-141 is a novel biomarker for metastatic colon cancer and predicts poor prognosis. *PLoS One*.

[68] Li LM, Hu ZB, Zhou ZX (2010). Serum microRNA profiles serve as novel biomarkers for HBV infection and diagnosis of HBV-positive hepatocarcinoma. *Cancer Research*.

[69] Duttagupta R, Jiang R, Gollub J, Getts RC, Jones KW (2011). Impact of cellular miRNAs on circulating miRNA biomarker signatures. *PLoS One*.

[70] McDonald JS, Milosevic D, Reddi HV, Grebe SK, Algeciras-Schimnich A (2011). Analysis of circulating microRNA: preanalytical and analytical challenges. *Clinical Chemistry*.

[71] Kirschner MB, Kao SC, Edelman JJ (2011). Haemolysis during sample preparation alters microRNA content of plasma. *PLoS One*.

[72] Sokoll LJ, Ellis W, Lange P (2008). A multicenter evaluation of the PCA3 molecular urine test: pre-analytical effects, analytical performance, and diagnostic accuracy. *Clinica Chimica Acta*.

[73] Kroh EM, Parkin RK, Mitchell PS, Tewari M (2010). Analysis of circulating microRNA biomarkers in plasma and serum using quantitative reverse transcription-PCR (qRT-PCR). *Methods*.

[74] Lee LW, Zhang S, Etheridge A (2010). Complexity of the microRNA repertoire revealed by next-generation sequencing. *RNA*.

[75] Ryan BM, Robles AI, Harris CC (2010). Genetic variation in microRNA networks: the implications for cancer research. *Nature Reviews Cancer*.

[76] Huang Z, Huang D, Ni S, Peng Z, Sheng W, Du X (2010). Plasma microRNAs are promising novel biomarkers for early detection of colorectal cancer. *International Journal of Cancer*.

[77] Bolstad BM, Irizarry RA, Åstrand M, Speed TP (2003). A comparison of normalization methods for high density oligonucleotide array data based on variance and bias. *Bioinformatics*.

[78] Deo A, Carlsson J, Lindlöf A (2011). How to choose a normalization strategy for miRNA quantitative real-time (QPCR) arrays. *Journal of Bioinformatics and Computational Biology*.

[79] Hua YJ, Tu K, Tang ZY, Li YX, Xiao HS (2008). Comparison of normalization methods with microRNA microarray. *Genomics*.

[80] Pradervand S, Weber J, Thomas J (2009). Impact of normalization on miRNA microarray expression profiling. *RNA*.

[81] Pepe MS, Etzioni R, Feng Z (2001). Phases of biomarker development for early detection of cancer. *Journal of the National Cancer Institute*.

[82] Simon R, Radmacher MD, Dobbin K, McShane LM (2003). Pitfalls in the use of DNA microarray data for diagnostic and prognostic classification. *Journal of the National Cancer Institute*.

